# Design and implementation of single switch integrated boost and flyback converter for renewable and sustainable energy

**DOI:** 10.1371/journal.pone.0287770

**Published:** 2023-06-30

**Authors:** R. Sathiya, M. Arun Noyal Doss

**Affiliations:** 1 Department of Electronics and Communication Engineering, College of Engineering and Technology, SRM Institute of Science and Technology, Vadapalani Campus, Chennai, India; 2 Department of Electrical and Electronics Engineering, SRM Institute of Science and Technology, Kattankulathur, Chennai, India; Galgotias University, INDIA

## Abstract

Renewable resources are being explored to meet the increasing energy demand in the world. The development of RES and their integration into the grid necessitate a voltage conversion to match with the grid voltage. This conversion can be implemented using DC-DC converters. A high-gain DC-DC Converter with low loss is proposed in this article. Thus, the proposed integrated converter is obtained by incorporating a boost converter at the primary side of the flyback converter (FLC) and a VM cell at the secondary side to perform a elevated voltage gain at a lower duty ratio. The Switched Capacitor network is implemented to elevate the voltage gain. The dynamic performance of a controller can be enhanced using an FOPID controller. A comparison analysis has been done using the most recent topologies in order to confirm the superiority of the Proposed converter. A 100W experimental prototype model has been constructed in order to further validate the simulation results. The efficiency of this converter is demonstrably significantly higher than the current topology, according to measured performance. Therefore, it can be said that this topology can be used for applications involving renewable and sustainable energy.

## Introduction

The need for energy also rises as a result of the rapid advancement of technology. The production of electricity relies heavily on fossil fuels almost everywhere in the world. However, the use of fossil fuels for power generation has declined due to a number of problems, such as air pollution and other environmental risks. Due to its greater availability, solar energy is the RES that is used the most frequently. Yet, partial shade situations may cause a decrease in the amount of electricity produced by PV. Traditional converters are often incorporated to overcome the drawbacks of low conversion efficiency and increased cost. A higher step-up DC-DC converter is used to convert the low voltage level to a higher voltage level for required applications [1, 2]. But due to a higher inrush current, the efficiency is reduced. The switching frequency of the system was raised to reduce the influence of inrush current and therefore the switches are subjected to high stress. The boost- type converters have a gain limitation because of their internal losses and also due to stress over the switches. It also has a negative effect on diodes, causing their efficiency to decline Hence, Converters with coupled inductance have been offered as a solution to this problem [3–6].

While switches are turned OFF, the leakage inductance develops a voltage spike. This in turn lowers the efficiency and causes high electromagnetic interference [EMI]. This can be overcome by active/passive clamped methods. Hence to override the above problems design of non-isolated boost converters were proposed [7–9].

However, it may leads to many disadvantages such as less reliability, more complexity and high cost. Moreover, the diode recovery issue is still a major issue in high gain applications [10–16]. Isolated Boost converters also exhibit higher voltage gain with a help of transformer. But the leakage inductance will leads to many problems like EMI etc., At the same time, the loss occurring in transformers will lead to high cost, low efficiency and more complexity in control strategy [17, 18]. The topology of the Boost converter with fly- back combination is designed to attain high voltage gain. This design is feasibly simple when compared to other existing converters. But, maintaining voltage balance among the capacitor is tedious [19].

The efficiency of the converter is significantly influenced by the diode’s reverse recovery loss. The reverse recovery loss can be decreased when the boost converter operates in critical current mode or on a discontinuous current model [20]. This will increase current ripples and stress in the input current. In order to defeat this, a bulky filter has to be implemented. Hence, it is not suitable for high-power applications [21]. Interleaved Boost Converters (IBC) can be employed for enhancement in both power level and density, with low thermal stress delivery. Thus, it exhibits less ripple current for a duty cycle ratio below 0.5 [22–24]. Conversely, it exhibits certain drawbacks, when implemented in PV-grid connected system. Simultaneously, voltage stress over the switches is high and hence, there will be a low efficiency. To achieve higher gain a Non-Isolated with diode capacitor is designed [25]. To attain this, VM units extended to ‘n’ stages. The level can be increased by increasing the duty cycle and VM units. Thus, it exhibits an efficiency about 93.6%.

A new converter using triple mode is invented [26], but it requires increased components to attain higher voltage gain. In practical utilization, its efficiency is about 93.86% at 500 W.

A novel converter called interleaved MBC with VM cells is developed. When load power is about 300W, its efficiency is 93.56%. The presence of diodes in this circuit results in a high loss.

In quasi-Z-source converters, an input current remains continuous and hence stress over the switches and capacitors is very low. But they do not exhibit high gain [27, 28].

A converter with higher gain and lower stress on switches was designed. Its efficiency is about 93.6% (@ 66 W). This converter exhibits high gain at a lower duty ratio. Its highest gain is above 12 times ideal conditions with a duty ratio 0.6. But in practice, it depends upon parasitic values of components and load [29].

Similar to step-down and step-up mode efficiency is 91.2% and 89%, respectively. The greatest efficiency, at an output current of 0.6 A while in boost mode, is 93.9 [30].

A new converter that exhibits continuous input current is suggested in [31]. However, at larger duty ratios, the voltage gain is restricted.

A single-stage AC/DC FLC with synchronous rectification(SR) function and three output windings is used in order to enhance the cross-regulation and efficiency [32]. Its maximum efficiency is 87%, around 3% greater than a traditional Schottky diode. This converter exhibits high gains in theory. Whenever blocking voltage occurs, it exhibits a significant reverse voltage over the switches. It is due to energy stored in its leaking inductance. As a result, dampening circuits are necessary, which increases costs and also reduces efficiency. Furthermore, a high gain ratio may cause the output diode to reach its higher peak voltage during commutations. In order to overcome the defect of the converter topology, hybrid combinations of converters are introduced [33].

A coupled inductor (CI) and a voltage multiplier (VM) are used without a high-duty cycle to increase ultra-high voltage gain. A regenerative passive clamp capacitor linked in parallel to the switch and the leaking inductor of the CI recycles the magnetic energy stored there, assisting in limiting the maximum voltage across the switch. Thus, it is recommended to uses a switch with low static drain to source ON-resistance to reduce conduction losses in switches and improves efficiency [34]. A flyback-forward DC–DC converter with zero current switching’s is suggested in this work. The efficiency is increased by using an auxiliary circuit without any additional switches to offer gentle switching conditions [35].

DC-DC “Switching” power converters are a common class of linear, time-variant devices that are challenging to model and simulate. They are created to change a source of direct current (DC) from one voltage level to another while keeping it stable within predetermined parameters. Because of parasitic characteristics a significant impact on how these circuits really operate in accurate time and frequency domain, simulation results depend on how well the converter circuit non-idealities are modelled [36].

Thus, in this work, a novel hybrid converter combining boost and FBC is implemented. The proposed converter features two outputs in series: a flyback type that symbolizes high gain and a boost type that makes use of the energy in the leakage inductance. The wide variety of conversion ratios and steady input current of the proposed IBFC converter are ideal for applications involving renewable energy.

The proposed converter advantages are

1. Elevated voltage gain
2. Switching stress is low
3. Higher Efficiency
4. Regulated output voltage

The above advantages are suitably confirmed by mathematical modelling, simulation using MATLAB/SIMULINK and Experimental prototype.

This article is structured as follows. The proposed system’s modelling is discussed in Section 2. A feasibility analysis of this converter is done in Section 3. Finally, Section 4 discusses a brief conclusion.

## Modeling of the proposed converter

### Converter

**Fig 1:**
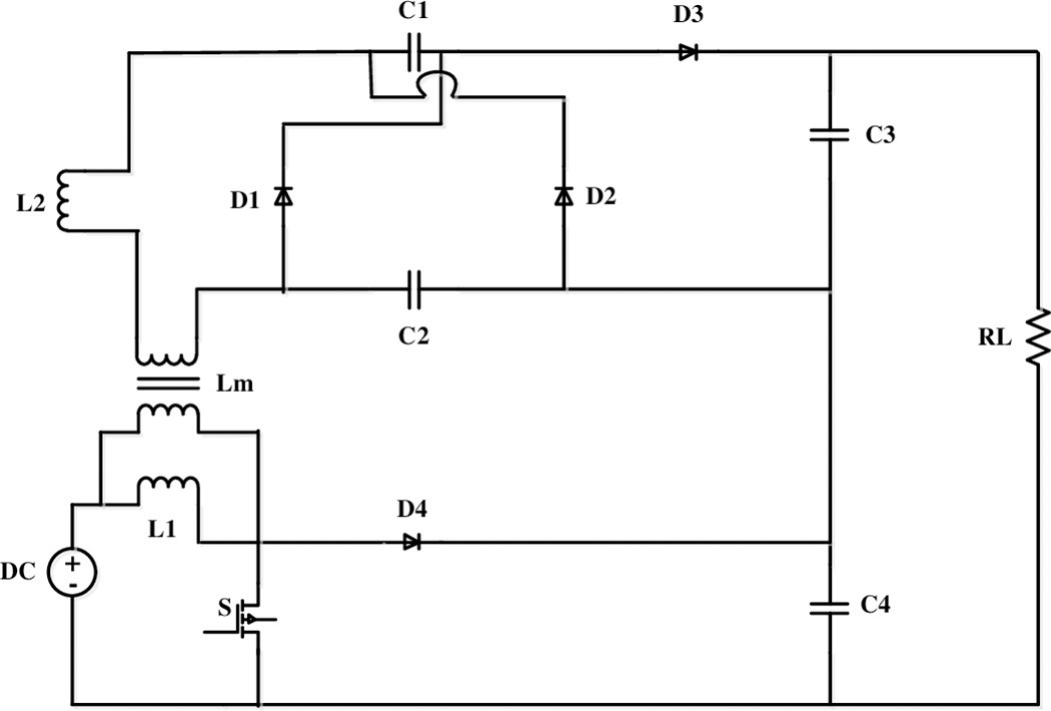
Representation of the proposed converter.

The proposed converter topology is shown in Fig 1, which is a hybrid of an FLC and a boost converter. A VM cell topology is incorporated in the converter to boost the voltage. It also reduces the di/dt stress on diodes.

**Fig 2:**
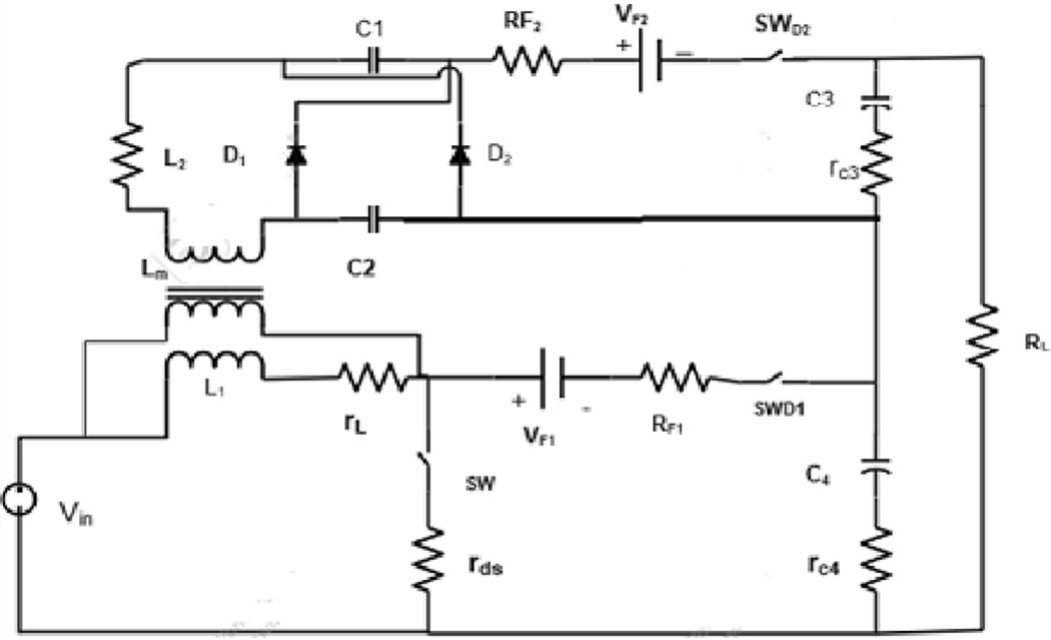
Proposed converter equivalent circuit.

Fig 2 depicts the proposed converter Equivalent circuit.

### Steady state examination

The assumptions are considered here in order to simplify the analysis of the proposed circuit.

1. The capacitors *C*_1_ and *C*_2_ are considered as same value. (*V*_*C*1_ = *V*_*C*2_ = *V*_*C*_);
2. The magnetising inductance (Lm) is taken into account as large and hence, the current across it remains constant.
3. Correspondingly, the voltage drop across the leakage inductor (*L*_2_), was neglected.

**Fig 3:**
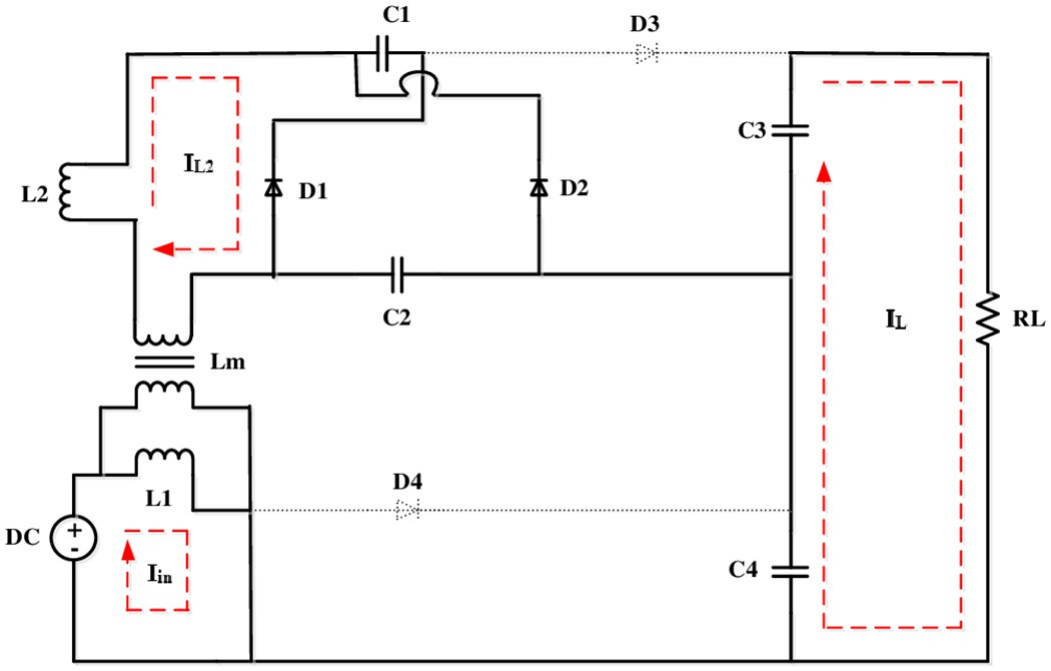
Mode 2.

**Fig 4:**
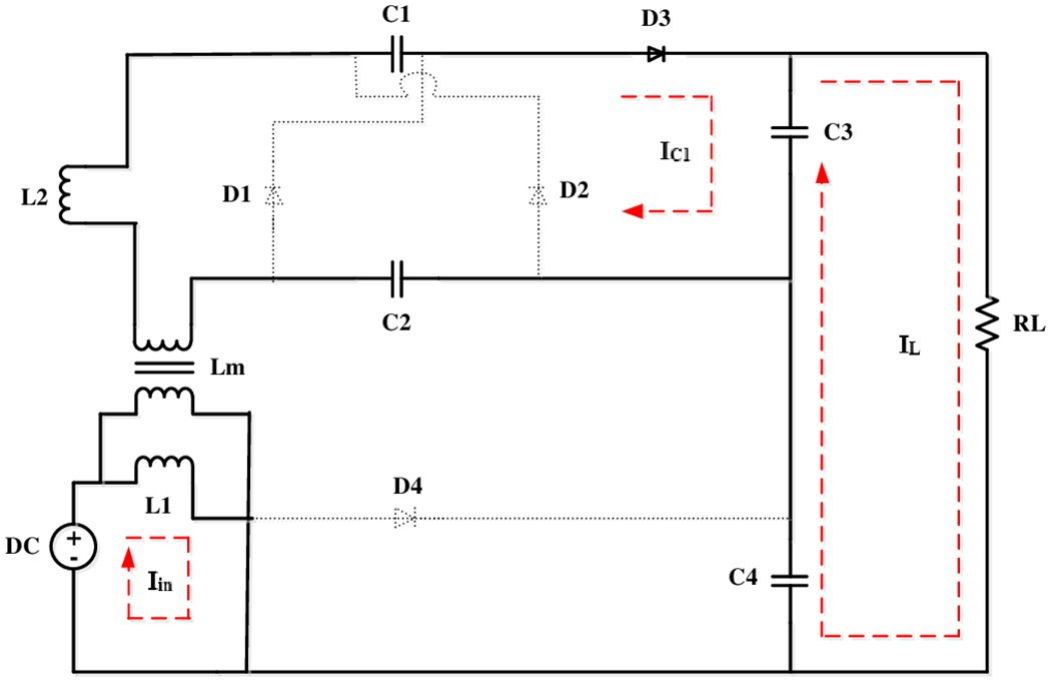
Mode 3.

**Fig 5:**
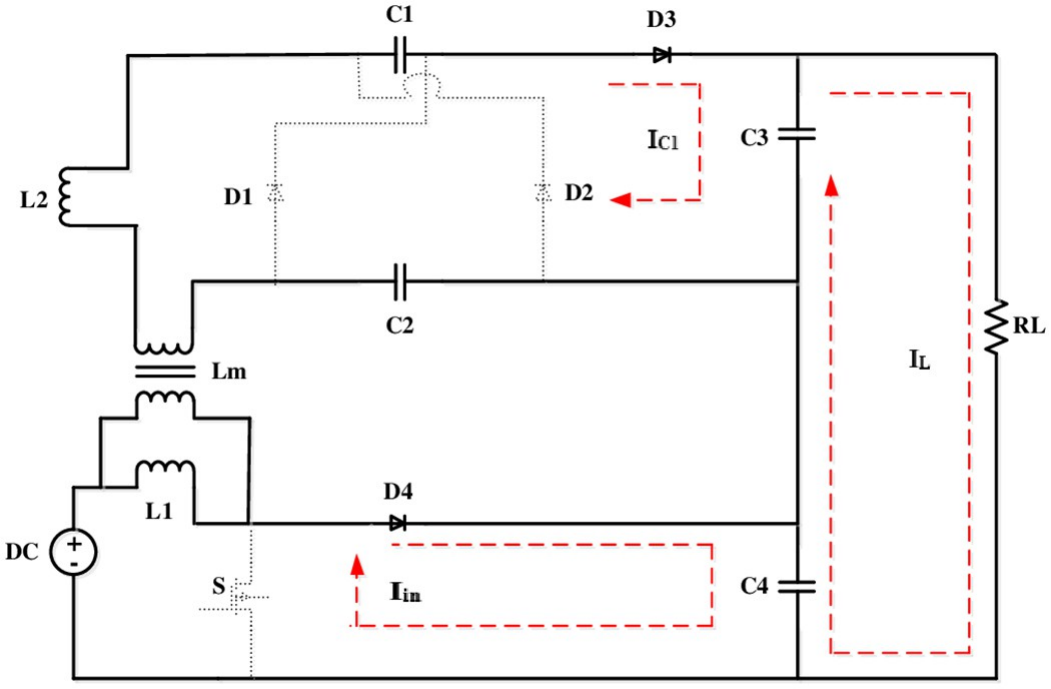
Mode 4.

**Fig 6:**
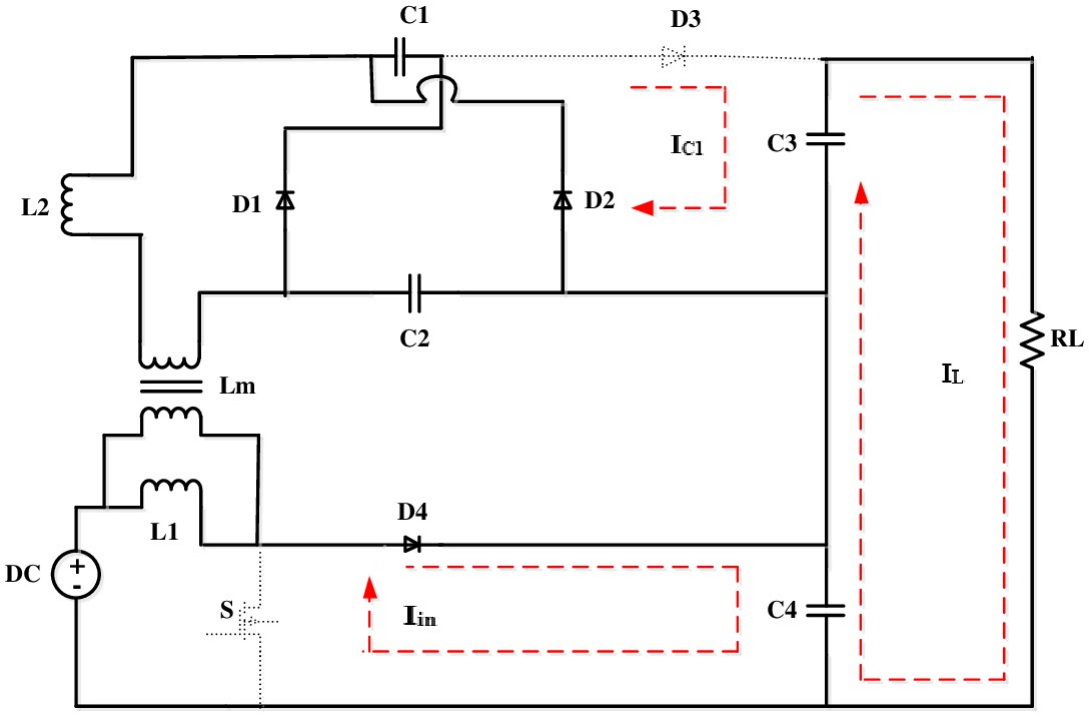
Mode 5.

The volt-sec balance principle is used to derive a converter’s voltage gain. The operating modes of this converter is depicted in Figs 3–6.

### Mode 1 (*t*_0_ − *t*_1_)

At this time (*t* = *t*_0_), all charges in the capacitors are negated and remains as ideal as Fig 1.

### Mode 2 (*t*_1_ − *t*_2_)

During *t* = *t*_1_, switch S is turned on at zero voltage. As a result, the current across *i*_*lm*_ is given as

$$\begin{array}{c} \frac{{di}_{lm}}{dt}=\frac{V_{1}+V_{c}/n}{L_{m}} \end{array}$$ (1)

Where,

n—Coupled Inductor turn’s ratio.

During S ON, current at the secondary side of CI starts charging SC (*C*_1_ and *C*_2_) in this circuit.

Thus, *i*_*l*2_, charges *C*_3_ and *C*_4_.

$$\begin{array}{c} \frac{{di}_{lm}}{dt}=\frac{V_{1}+2V_{c}/n}{L_{m}} \end{array}$$ (2)

Because *i*_*l*2_ is negative at that point, its magnitude begins to diminish. At this condition, the operation of the circuit is shown in Fig 3.

### Mode 3 (*t*_2_ − *t*_3_)

In this Fig 4, *i*_*l*2_ approaches zero at this point. ie. *D*_1_ and *D*_2_ remains turn off. This reduces the reverse recovery problems. As a result of shift in *i*_*l*2_, *C*_1_ and *C*_2_ discharges to *C*_3_. As a result, the current through the inductor *i*_*m*_ is shown as

$$\begin{array}{c} \frac{di_{lm}}{dt}=\frac{V_{1}+\left(2V_{c}-V_{c}3\right)/n}{L_{m}} \end{array}$$ (3)

### Mode 4 (*t*_3_ − *t*_4_)

S is turned off during *t* = *t*_3_. *V*_*C*4_ is charged by the current flowing across *i*_*m*_ and is depicted in Fig 5.

Due to the fact that *V*_*O*_ is greater than *V*_*C*4_, the voltage stress on S is relatively modest. As a result, no additional clamp circuit is required.

During this interval, the current (*i*_*m*_) is derived as

$$\begin{array}{c} \frac{{di}_{lm}}{dt}=\frac{V_{1}-V_{c4}+\left(2V_{c}-V_{c3}\right)/n}{L_{m}} \end{array}$$ (4)

### Mode 5 (*t*_4_ − *t*_5_)

In this Fig 6, *i*_*l*2_ is reversed and *i*_*m*_ is

$$\begin{array}{c} \frac{{di}_{lm}}{dt}=\frac{V_{1}-V_{C4}+V_{b}/n}{L_{m}} \end{array}$$ (5)

### Mode 6 (*t*_5_ − *t*_6_)

The current *i*_*m*_ remains same as it was in the preceding mode at *t* = *t*_5_.

**Fig 7:**
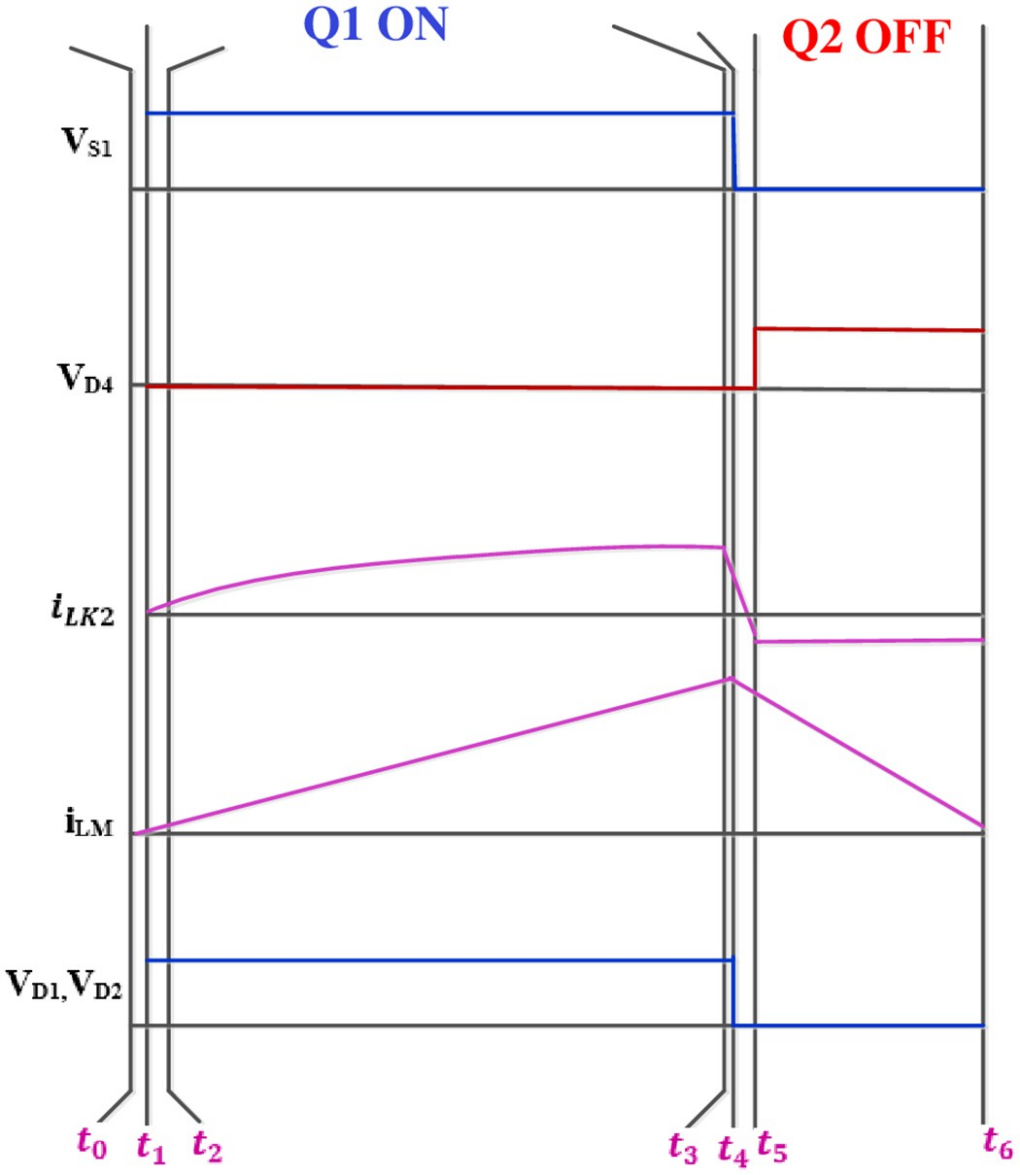
Modes of operation.

Fig 7 depicts the different modes of operation of the converter.

### Analysis and design considerations

#### Voltage gain

The converter’s output voltage *V*_*o*_ can be computed as

$$\begin{array}{c} V_{o}=V_{C3}+V_{C4} \end{array}$$ (6)

The association of *V*_1_, *V*_*O*_, *V*_*C*3_, *V*_*C*_ and *V*_*C*4_ may then be obtained using voltage second balancing analysis over (*L*_*m*_ and *L*_2_) in modes 3 and 6.

$$V_{C4}=\frac{1}{1-D}V_{1}$$ (7)

$$V_{c}=\frac{D}{1+D}V_{C3}$$ (8)

Then by rearranging Eqs (3), (6), (7) and (8) the gain of this converter can be derived as,

$$\begin{array}{c} M=\frac{V_{o}}{V_{1}}=\frac{1+n+nD}{1-D} \end{array}$$ (9)

According to the preceding equation, By varying turns ratio of mutual inductance, the gain can be altered.

### Voltage stress analysis

Due to the fact that *V*_*O*_ is greater than *V*_*C*4_, voltage stress over S is relatively small. Similarly, all diode’s voltage stress (*D*_1_, *D*_2_) is determined by *V*_*C*3_ and *V*_*C*_.

A current stress across diode *D*_3_ can be expressed as,

$$\begin{array}{c} I_{D1_{PEAK}}=\frac{2V_{0}}{DR} \end{array}$$ (10)

Similarly, the current stress across *D*_1_ and *D*_2_ is given by as,

$$\begin{array}{c} I_{D2_{PEAK}}=\frac{V_{0}}{\left(1-D\right)R} \end{array}$$ (11)

To examine the coupling factor influence, a specific analysis was performed on a magnetic coupled inductor based boost converter. High voltages can be obtained by utilizing a coupled inductors. In reality, as the insulation voltage and fabrication limitations increase, the coupling factor drops.

### Design of inductors

The inductor’s turn ratio is critical for the converter’s voltage gain. As a result, the turns ratio should be chosen in such a way that it influences the inductor’s loss and size. The magnetic core’s non-saturation restriction could be utilised to calculate the minimum number of turns on primary side

$$\begin{array}{c} B_{s}n_{0}A_{e}>E_{i}T_{n} \end{array}$$ (12)

where,

Bs-Flux density

*n*_0_–No. of turns on primary side

*A*_*e*_-Cross section (Magnetic Core).

*E*_*i*_*T*_*n*_-Maximum volt-second (Primary Side).

A lower turns ratio indicates

a) low copper and winding loss

b) A smaller coupled inductor. As a result, a low ratio of turns is preferred.

Similarly, the inductors namely *L*_*m*_ and *L*_*k*1_ should be selected in such a way to reduce the di/dt stress over the diodes.

In designing, the selection of proper rating for all the components in the converter is essential. More importance is therefore given for designing the converter components. While designing an inductor, the inductor ripple current(I_*L*_) must be considered. Thus, it can be determined using a formula

$$\Delta (I_{L})=30\%\times (\frac{I_{in}}{\eta })$$ (13)

$$\Delta (I_{L})=0.3\times (\frac{P}{Vin\times \eta })$$ (14)

$$L_{1}=(\frac{V_{in}\times \left(D\right)}{20\times \Delta \left(I_{L}\right)\times \left(F_{sw}\right)})$$ (15)

Where *L*_1_ = *L*_2_

### Design of capacitors

$$\begin{array}{c} C_{3}=\frac{I_{out}\times \left(D_{max}\right)}{\Delta V_{c0}\times \left(F_{sw}\right)} \end{array}$$ (16)

Assumed *C*_3_ = *C*_4_.

Voltage Multiplier cells: *C*_1_&*C*_2_

$$\frac{\Delta V_{c0}}{V0}=\frac{n}{4DR_{0}C_{4}F_{sw}}$$ (17)

$$C1,C2=\frac{n\times V0}{4DR_{0}\Delta V_{c0}F_{sw}}$$ (18)

### Efficient and transient analysis

The following equations are used to calculate the theoretical loss of the proposed topologies. Conduction losses of passive elements, conduction and switching losses of diodes and power semiconductor are taken into consideration.

$$\begin{array}{c} P_{Total.loss}=P_{switch}+P_{diode}+P_{inductor}+P_{capacitor} \end{array}$$ (19)

Conducting losses of switches

$$\begin{array}{c} P_{CS1}=\frac{1}{T_{s}}\int_{0}^{DT_{s}}\left(V_{s1}i_{s1}+r_{s1}i_{s1}^{2}\right)dt \end{array}$$ (20)

Conducting losses of diodes can be derived from the following equation

$$\begin{array}{c} P_{CD1}=\frac{1}{T_{s}}\int_{0}^{DT_{s}}\left(V_{D1}i_{D1}+r_{D1}i_{D1}^{2}\right)dt \end{array}$$ (21)

Switching losses of switches

$$\begin{array}{c} P_{SS1}=\frac{1}{T_{s}}\int_{0}^{DT_{s}}V_{s1}i_{s1}dt \end{array}$$ (22)

Switching losses of diodes can be derived from the following equation

$$\begin{array}{c} P_{SD1}=\frac{1}{T_{s}}\int_{0}^{{DT}_{s}}V_{D1}i_{D1}dt \end{array}$$ (23)

Therefore, both the switching and conduction losses in the switch and diode are expressed as,

$$\begin{array}{c} P_{SW}=(\frac{{V_{0}}^{2}}{{R_{0}}^{2}}{\left(\frac{\left(2-D_{1}\right)}{\left(1-D_{1}\right)}\right)}^{2}DR_{ds(on)})+\frac{f_{s}}{2}\left[t_{r}+t_{f}\right]I_{sw}V_{swm} \end{array}$$ (24)

Diode loss considering diode voltage and resistance is given below and reverse recovery loss (*PD*_*sw*_) is small contributor and therefore neglected,

$$P_{D}=V_{f}I_{D(avg)}+I_{D(rms)}^{2}R_{f}$$ (25)

$$P_{Dsw}=V_{r}Q_{rr}f_{s}$$ (26)

Similarly, the power loss and capacitor loss are given as

$$P_{L}=I_{L(rms)}^{2}r_{L}$$ (27)

$$P_{C}=I_{C(rms)}^{2}r_{C}+I_{C0(rms)}^{2}r_{C0}$$ (28)

$$P_{T}=P_{SW}+P_{D}+P_{L}+P_{C}$$ (29)

$$\eta =\frac{P_{0}}{P_{0}+P_{T}}$$ (30)

Total Loss

$$\begin{array}{cc} P_{T} & =9.958W\\ \eta & =\frac{100}{100+9.958}\\ & =90.94\% \end{array}$$

**Fig 8:**
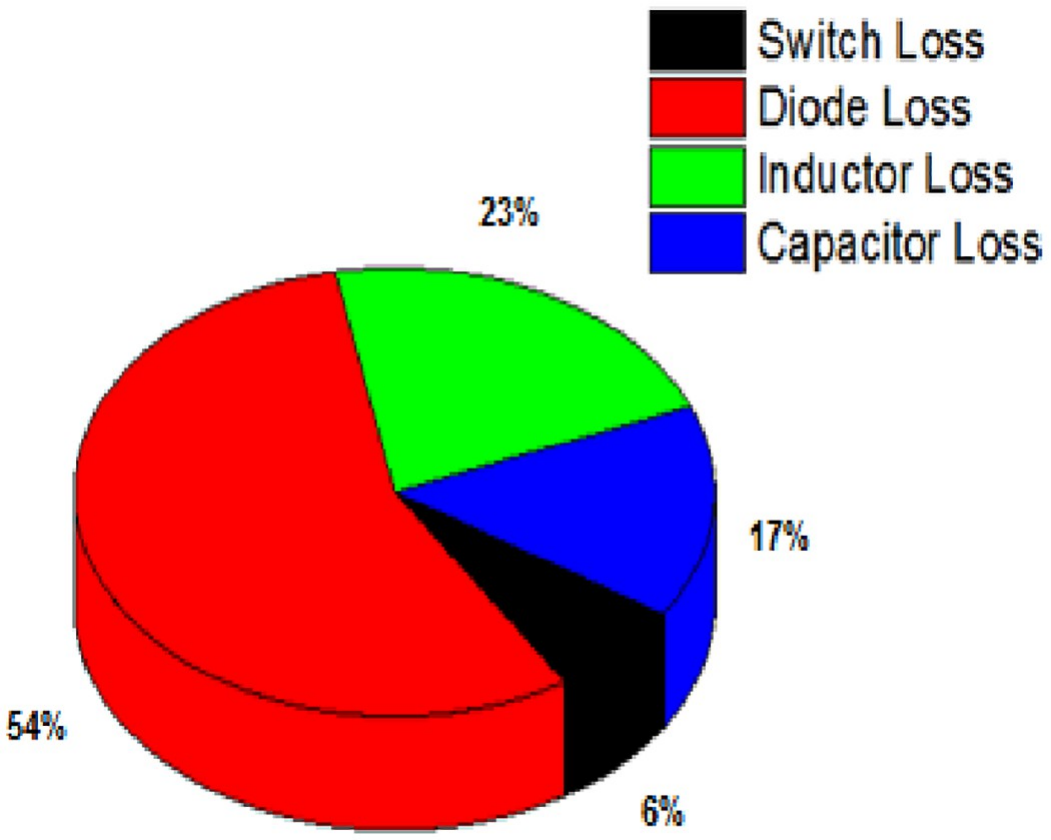
Losses of the proposed topology.

Fig 8 depicts the percentage of losses occurred due to various components in this. The converter’s efficiency is about 90.95%. As this converter has more number of diodes, diode loss is significantly high in this proposed architecture.

### Voltage ripple

The ripple voltage on *V*_*C*_ can be derived as,

$$\begin{array}{c} \frac{{\Delta V}_{c}}{V_{0}}=\frac{1}{{RC}_{c}f} \end{array}$$ (31)

where,

Cc—switch capacitance, C1 and C2.

According to the Eq (31), a large capacitance is required to overcome the voltage ripple problem. As a result, Cc can be calculated based on the desired voltage ripple. It also applicable for output capacitors.

### State space averaging and modeling

The supportive parameters, as shown in Fig 1, independent state variables and the state space vector is expressed as follows:

$$\begin{array}{c} \left\lfloor x\left(t\right)\right\rfloor =|\begin{array}{c} i_{lm}\left(t\right)\\ v_{0}\left(t\right) \end{array}| \end{array}$$ (32)

This equation represents the system output in terms of current state and input, forms a state space equation of the system which can be represented as

$$\begin{array}{cc} \dot{X(t)} & =AX(t)+BU(t)\\ Y(t) & =CX(t)+DU(t) \end{array}$$

Thus, it can be also represented as

$$\begin{array}{cc} \left[\begin{array}{c} \frac{di_{1}}{dt}\\ \frac{di_{2}}{dt}\\ \frac{dV_{c1}}{dt} \end{array}\right] & =[\begin{array}{ccc} 0 & 0 & 0\\ 0 & 0 & 0\\ 0 & \frac{-1}{C_{1}} & 0 \end{array}][\begin{array}{c} i_{1}\\ i_{2}\\ V_{c1} \end{array}]\\ & \;+[\begin{array}{c} \frac{1}{DL_{1}}-(\frac{1}{L_{1}})*\{1+(\frac{Ton}{Toff})N\}-(\frac{1}{L_{1}})(\frac{1}{1-D})\\ (\frac{1}{DL_{2}})-(\frac{1}{L_{2}})*\{1+(\frac{Ton}{Toff})N\}\\ \left(\frac{1}{RDC_{1}}\right) \end{array}]\left[V_{i}\right]\\ Y & =\left[0\;0\;1\right][\begin{array}{c} i_{1}\\ i_{2}\\ V_{e1} \end{array}]+\left[0\right]\left[Vin\right] \end{array}$$

### Controller implementation

#### FOPID controller

Fractional PID controllers have been utilized to enhance the system control in industrial applications. It is a feedback controller that can be utilized in a various applications. As it has three parameters in its structure, it is referred as three modes controller.

The goal of the developed controller is to reduce integral square error and hence boost dynamic response. The values of the three action’s parameters can be depicted in terms of time:

P—Current error,
I—Addition of prior errors
D—Forecast of future errors.

**Fig 9:**
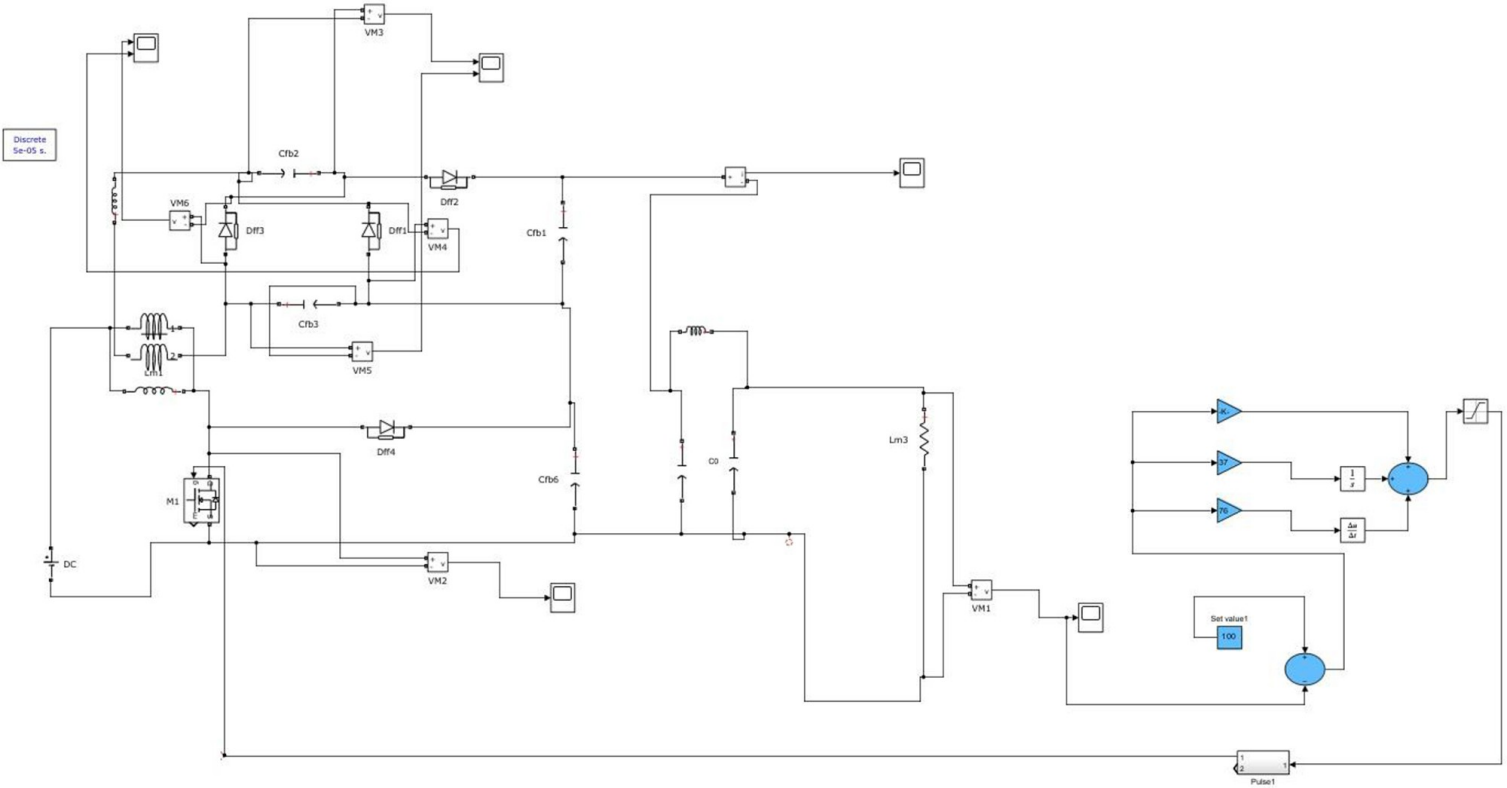
Closed loop FOPID controller using MATLAB/Simulink model.

A Fractional calculus based classic PID Controller is shown in Fig 9.

The gain constant *K*_*i*_ and *λ* can be obtained from the equations, and then, *K*_*p*_ can be obtained. After finding *α*, the parameters of the simplified FOPID controller can be obtained using the above discussed specifications. Fig 9 depicts Closed loop model of FOPID Controller using MATLAB/Simulink model.

The transfer function of *PI*^λ^*D*^*μ*^ can be depicted as,

$$\begin{array}{c} C\left(S\right)=\frac{U(S)}{E(S)}=K_{p}+K_{i}S^{-\lambda }+K_{d}S^{\mu } \end{array}$$ (33)

Where

λ & *μ*—Real numbers (Positive)

*K*_*p*_, *K*_*i*_ & *K*_*d*_—Gain constants.

While substituting λ and *μ* = 1, the Eq (33) becomes PID controller and the controller acts like a PID controller.

Similarly, by substituting λ = 1, *μ* = 0, PI can be obtained.

When λ = 0, *μ* = 1, it behaves like a PD controller.

## Results and discussion

The open loop and closed loop performance of the proposed converter is verified using MATLAB/SIMULINK Model. Table 1 lists the components utilized in the converter.

**Table 1:** Design parameters of converter.

Components	Parameters
*V*_1_	18 V
*V*_*o*_	220 V
*C*_1_, *C*_2_	36 *μ*F
*C*_3_, *C*_4_	47 *μ*F
n	3.8
*L*_*m*_	617.23 *μ*H
*L*_*k*2_	17.12 *μ*H
*fs*	16 kHz
*D*	0.63

**Fig 10:**
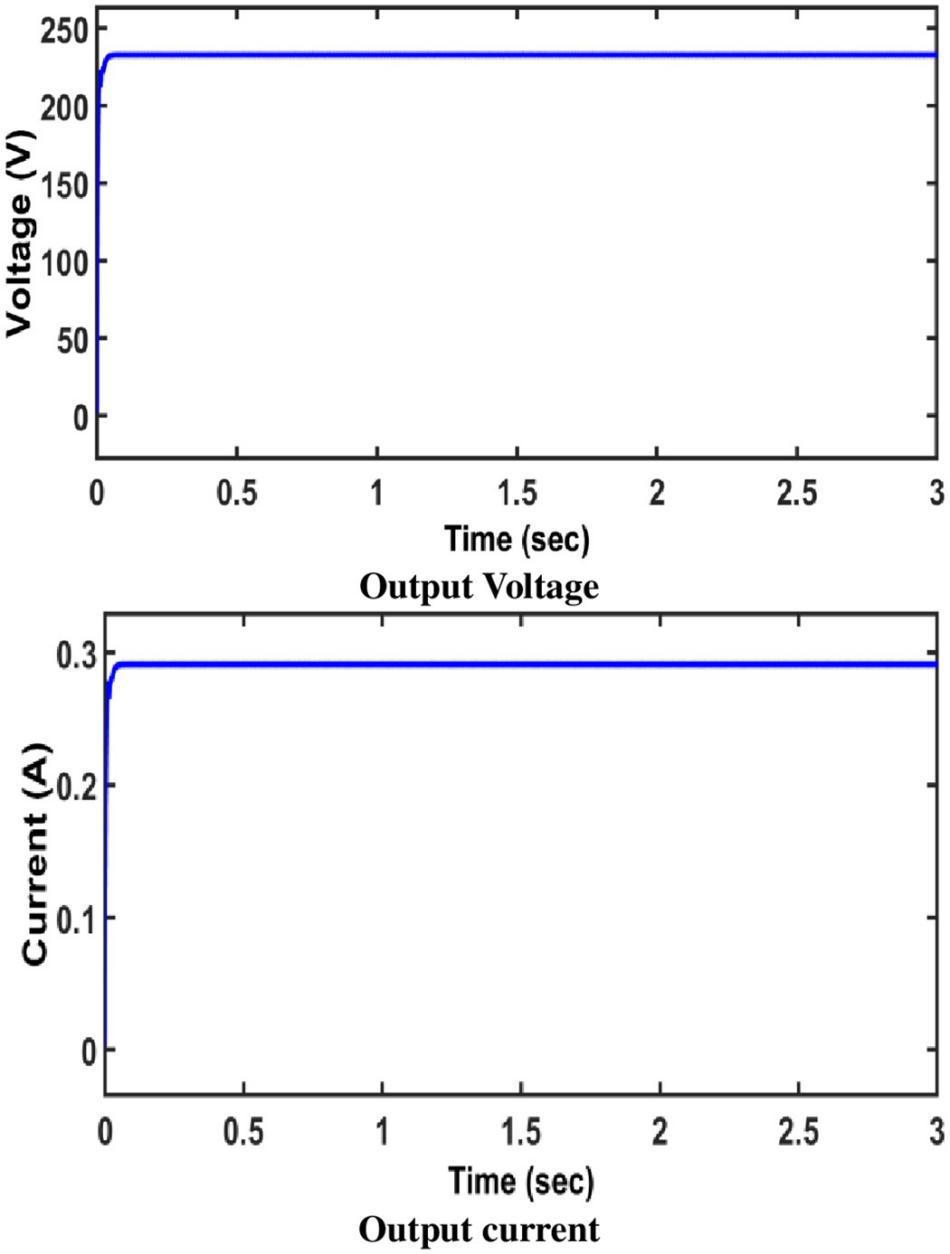
Output voltage /current (open loop).

The open loop converter output voltage and current is portrayed in Fig 10.

**Fig 11:**
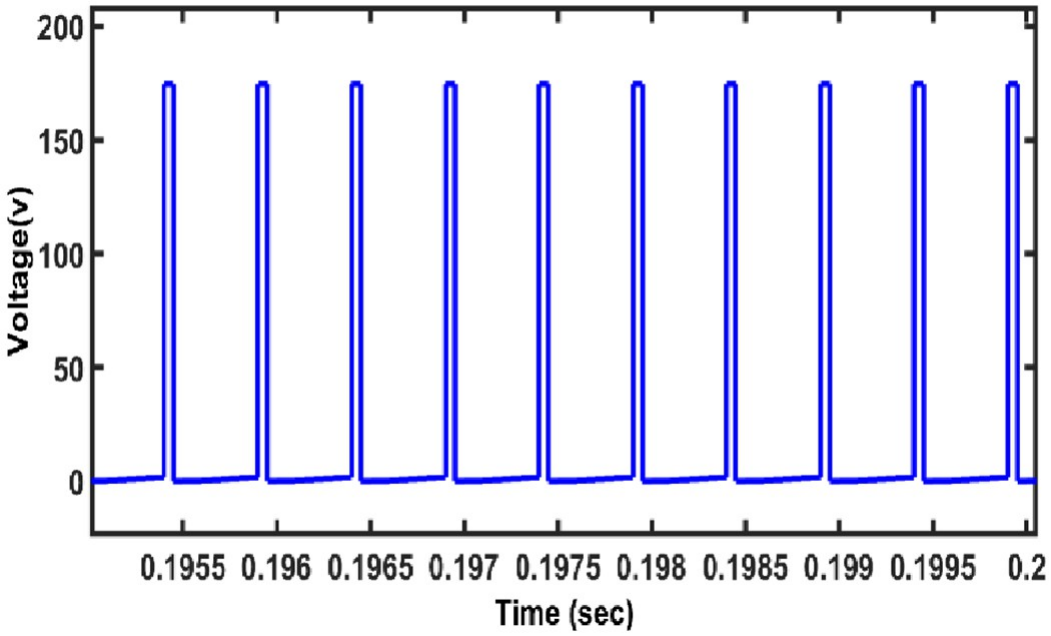
Voltage across the switch.

Voltage stress over the switch is presented in Fig 11.

**Fig 12:**
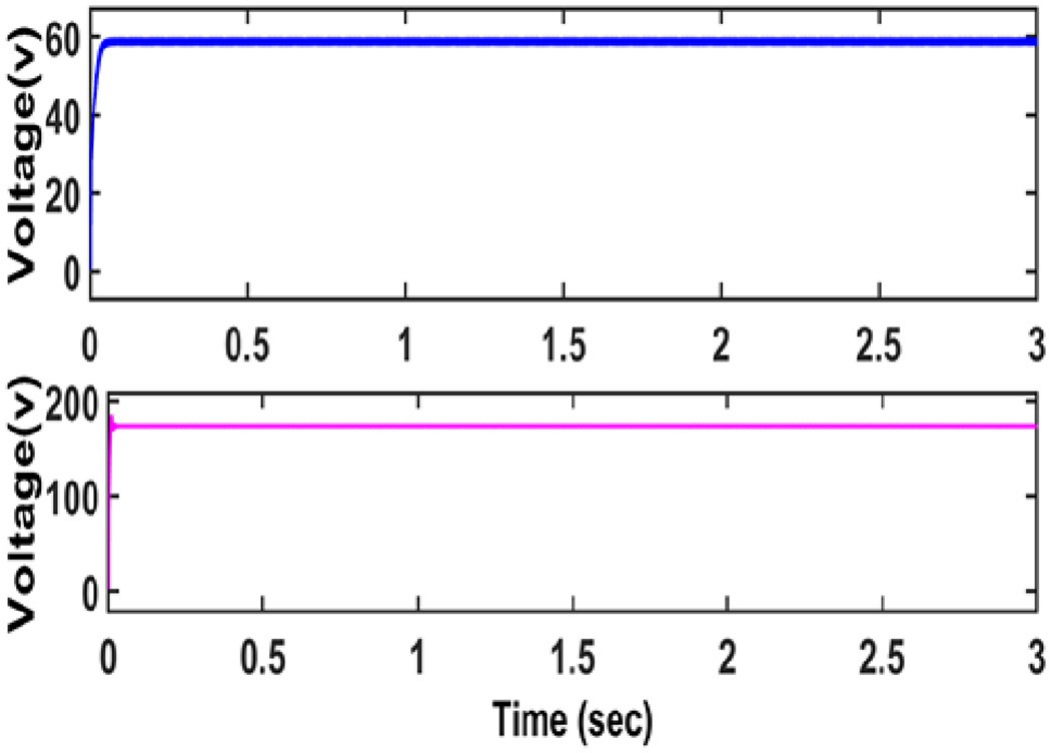
Voltage across C3 and C4.

The voltage across the MOSFET switch is an average of 150 V, which is lower than that of the output voltage. The average voltage on VM cell diodes such as D1, D2 is 20V. Hence, it is concluded that as voltage stress over the switch remains low ensures high efficiency. The voltage across the capacitor C3 and C4 is depicted in Fig 12.

**Fig 13:**
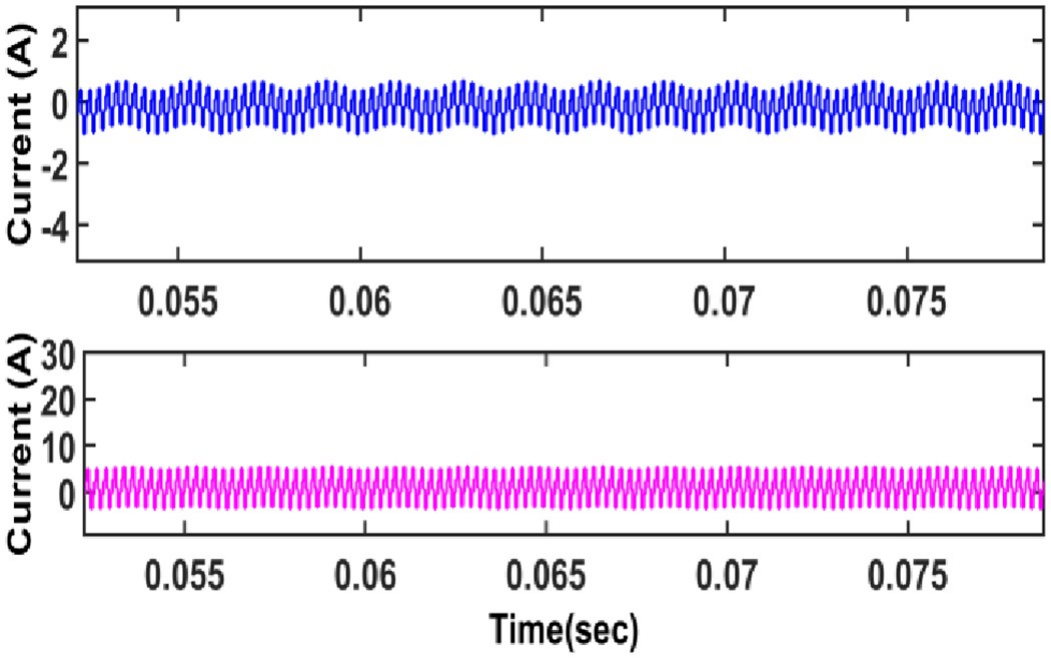
Current across inductor.

The current across the inductor *L*_1_ and *L*_2_ is depicted in Fig 13.

Fig 13 depicts the current across the primary and secondary side of a CI. From the figure, it is observed that whenever primary coil of coupled inductor is energized, secondary coil receives energy due to mutual inductance between coupled inductor.

**Fig 14:**
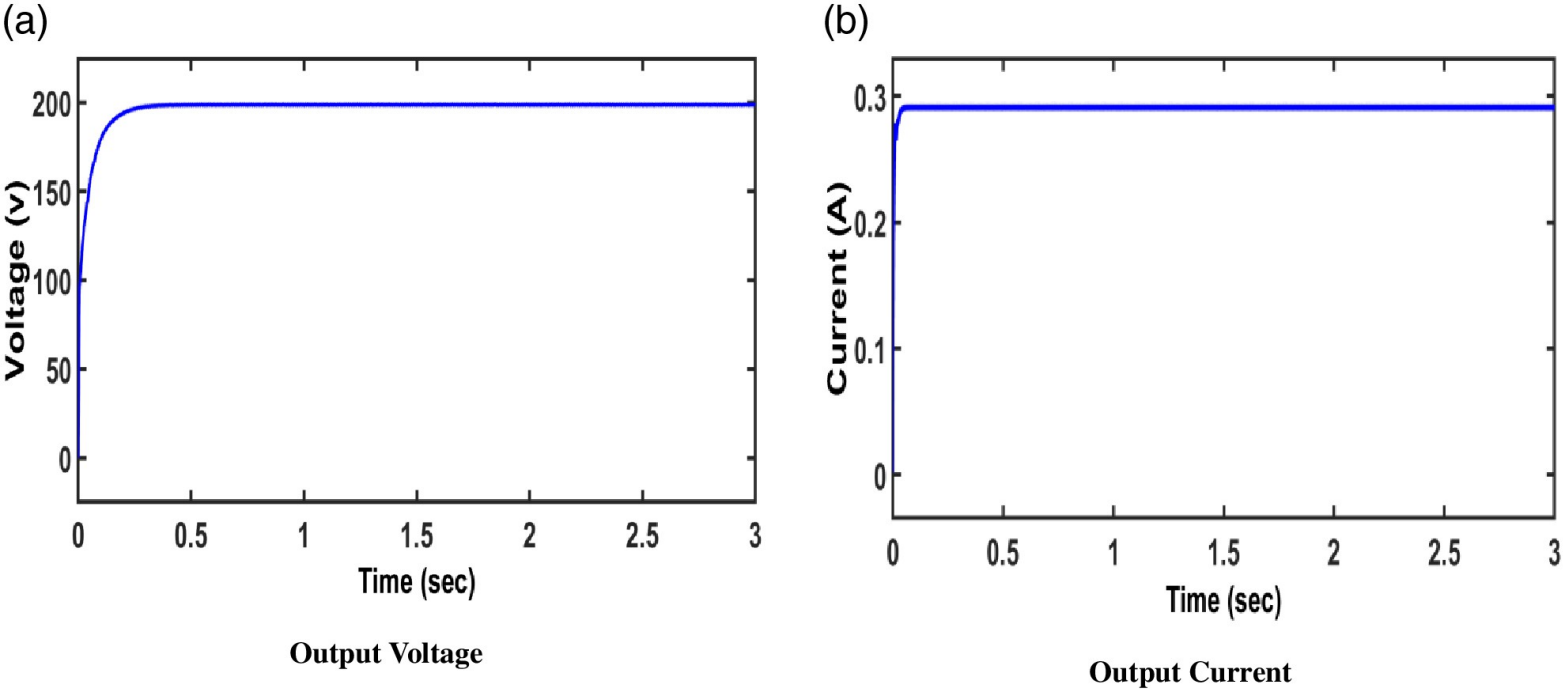
Output voltage /current of the converter (FOPID controller).

Thus, an analysis of this converter under closed loop mode is portrayed in Fig 14. In this closed loop study, to ensure the system’s stability, a FOPID controller is implemented. Regardless of variations in input voltage/load, it maintains a constant voltage at the output.. The gains of the converter are determined using ZN method.

Parameter settings considered for FOPID controller are as follows

*K*_*p*_ = 0.5010,

*K*_*i*_ = 4.8940,

*K*_*d*_ = 4.8940,

λ = 1 and

*μ* = 0.99.

There is less output variability when compared to open loop control. The results reveal that the FOPID controller is more efficient than a typical controller.Both open loop and closed loop test of the proposed converter show higher voltage gain.

**Fig 15:**
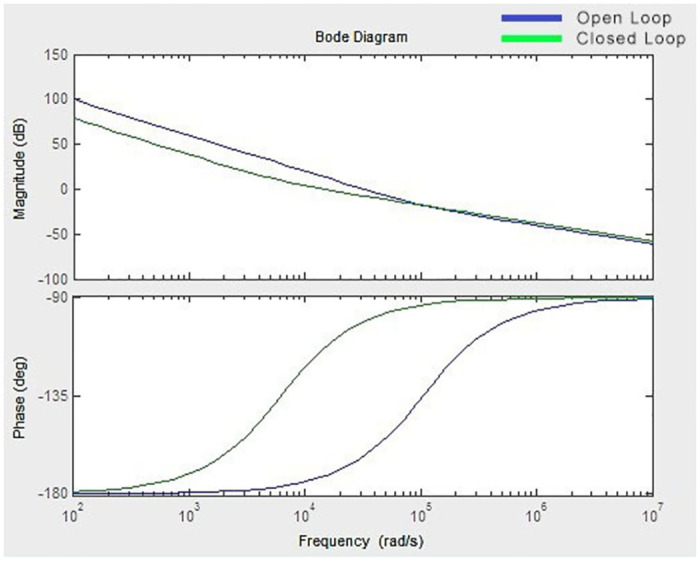
Bode plot.

Thus, to analyse the effect of stability of the converter, bode plot analysis have been carried out. Fig 15 depicts the bode plot diagram of the proposed converter.

The value of gain and phase margin is observed from the bode plot (Gm = 1.1657e^−11^, Pm = 2.0149 for open loop; Gm = 3.0215e-16, Pm = 9.6520 for closed loop) and are positive. Hence the proposed converter remains stable and controllable in closed loop also.

### Performance analysis of the converter with PV module

**Fig 16:**
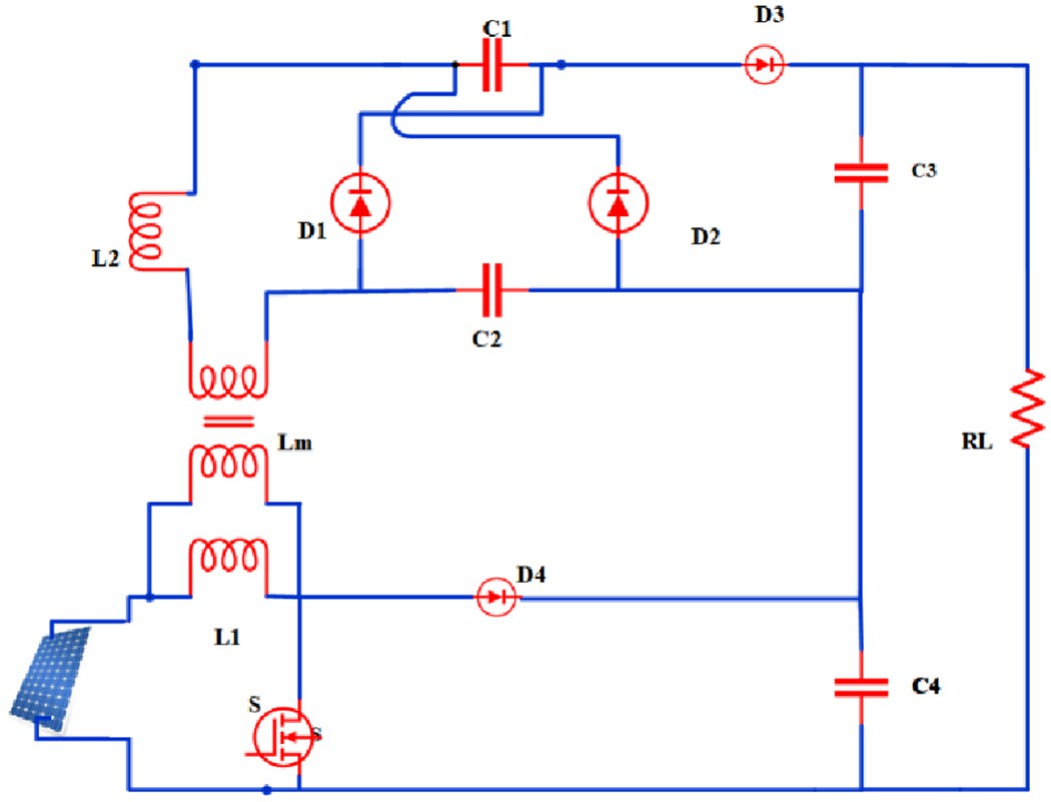
Proposed converter with PV panel.

Fig 16 depicts the PV integrated proposed converter.

Thus, the PV modelling for the proposed configuration is depicted below

The solar cell is the basic component of PV arrays. the P-N junction that transforms light energy into electrical current. When it comes into contact with light, the photons are absorbed. Only photons with energies bigger than the energy gaps are absorbed, hence the absorbed photons create electron-hole pairs.

The electric fields are influenced by this method of photons with electron hole pairs, which also produces current that is proportionate to solar radiation.

**Fig 17:**
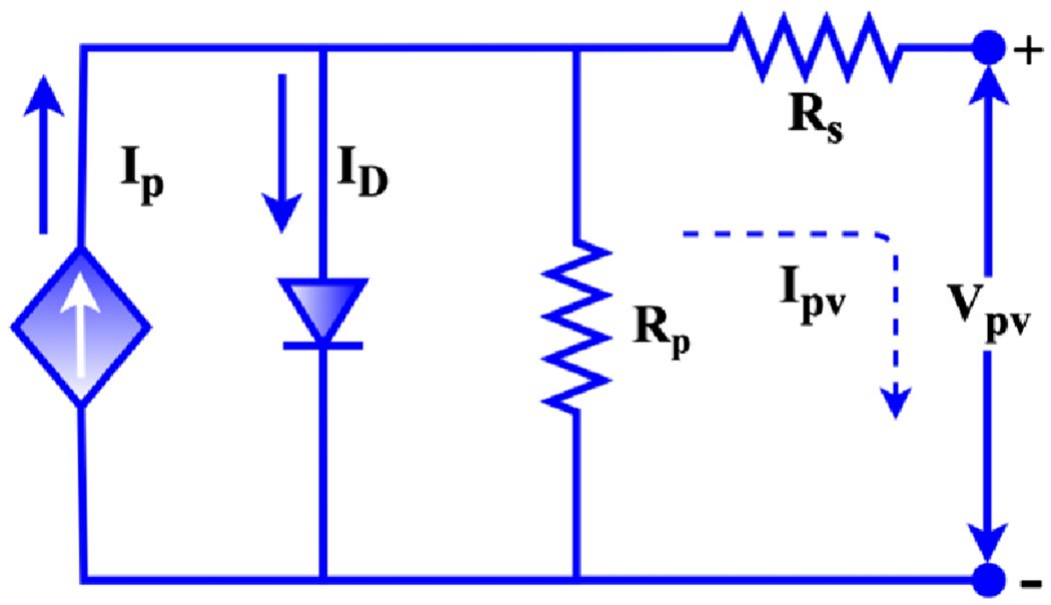
Equivalent circuit of a PV cell.

The PV cell Equivalent circuit model is stated in Fig 17.

The PV module is formed by connecting numerous PV cells. PV modules are connected in series and parallel to create the PV array. The following equation represents a PV array as a mathematical model.

$$\begin{array}{cc} I & =n_{p}I_{ph}-n_{p}I_{s}[exp(\frac{q}{KTA})*\left(V/n_{s}\right)-1]\\ I & =n_{p}I_{ph}-n_{p}I_{rs}[\text{exp}\;(\frac{q}{KTA})*\left(V{/}_{n_{s}}\right)-1] \end{array}$$ (34)

where

*I*_*ph*_—Photo current

*I*—output current PV array

*V*—output voltage PV array

*n*_*s*_—series number of cells

*n*_*p*_—parallel number of cells

*q*—charge

*K*—Boltzmann’s constant [8.62 × 10^−5^ eV/K]

*A*—p-n junction Ideality Factor. It ranges between 1–5.

*T*—cell temperature (K)

*I*_*rs*_—reverse saturation current.

Thus the PV power can be calculated using:

$$\begin{array}{c} P=IV=np\;Iph\;V[(q/KTA)*(V/ns)-1] \end{array}$$ (35)

The irradiance, environmental conditions and the currents drawn out of the cells optimizes the power emitted by the PV systems. Few applications demands power more than the offered limit of the PV systems. The appliances like usage of power grid battery charging or electric motor usage etc. demands more power from the PV system. In these cases the power from the PV system has to be maximized which could be done using the power conversion system.

**Fig 18:**
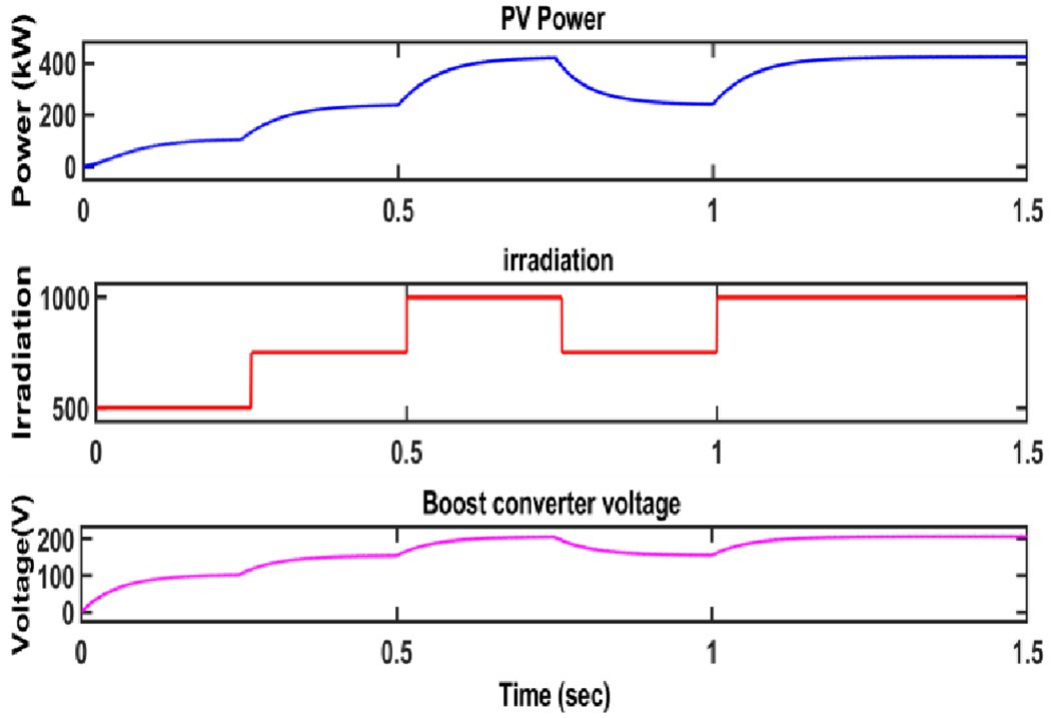
Converter voltage and PV power with respect to varying irradiance.

Fig 18 depicts the performance of the converter under different irradiance condition.

**Fig 19:**
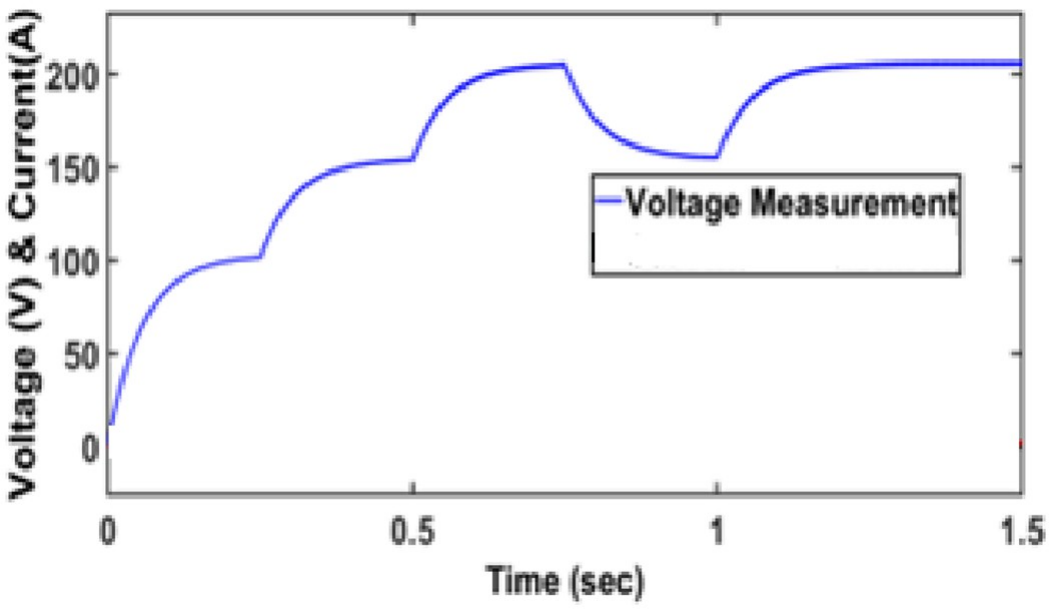
Voltage obtained from the converter.

Fig 19 shows the voltage of the proposed converter.

## Comparative analysis

The formulated topology is compared with several other recent converter structures in this section. In order to demonstrate the converter’s benefits, it is compared to other high step-up converters that have been shown. Table 2 demonstrates that the proposed configuration enables semiconductors with a minimum number of components constant input current and soft switching circumstances.

**Table 2:** Performance comparison.

Topology	Voltage gain	Voltage stress switches	No of Switches	No of Diodes	No of Inductors	No of Capacitors	Switching State	No of Total Count	Efficiency
**High gain Converter (2020)** [37]	$\frac{\left(1+D\right)}{\left(1-D\right)}$	$\frac{\left(Vo-Vin\right)}{2},Vo$	2	3	2	1	ZVS	8	89%
**Hybrid switched inductor (2015)** [38]	$\frac{\left(1+3D\right)}{\left(1-D\right)}$	$\frac{(1+G)Vi}{2}$	2	4	3	1	ZVS	10	90.3%
**Relift converter (2018)** [39]	$\frac{\left(M+1\right)}{{\left(1-D\right)}^{2}}$	$\frac{\left(Vg\right)}{\left(1-D\right)},\frac{\left(Vg\right)}{{\left(1-D\right)}^{2}}$	2	4	2	4	ZVS	12	90.5%
**ZVT interleaved boost dc/dc converter** [40]	$\frac{1}{\left(1-d\right)}$	1	3	6	-	1	ZVS	10	87%
**Walton transformerless high step-up dc-dc converter** [41]	$\frac{2m}{\left(1-d\right)}$	$\frac{1}{2m}$	4	2m	-	2m	-	4+4m	87.5%
**ZV-ZC High gain DC-DC Converter** [42]	$\frac{\left(2+N\right)}{\left(1-d\right)}$	$\frac{1}{\left(2+N\right)}$	2	4	-	5	ZVS	11	90%
**Single Switch High gain dc dc converter** [43]	$\frac{\left(3-D\right)}{{\left(1-D\right)}^{2}}$	$\frac{2}{{\left(1-D\right)}^{2}}$	1	6	3	6	ZVS	16	88%
**Proposed**	$\frac{1+n+nD}{1-D}$	*V*_*C*4_	1	4	2	4	ZVS	11	90.95%

The gain of the converter is increased by using a CI in it. i.e., as shown in the table above, more than ten times as much as a normal converter. This can be utilised in Applications of PV Systems. The voltage across the switch in this converter is fairly modest when compared to a boost converter. The proposed converter has decreased conduction loss.

**Fig 20:**
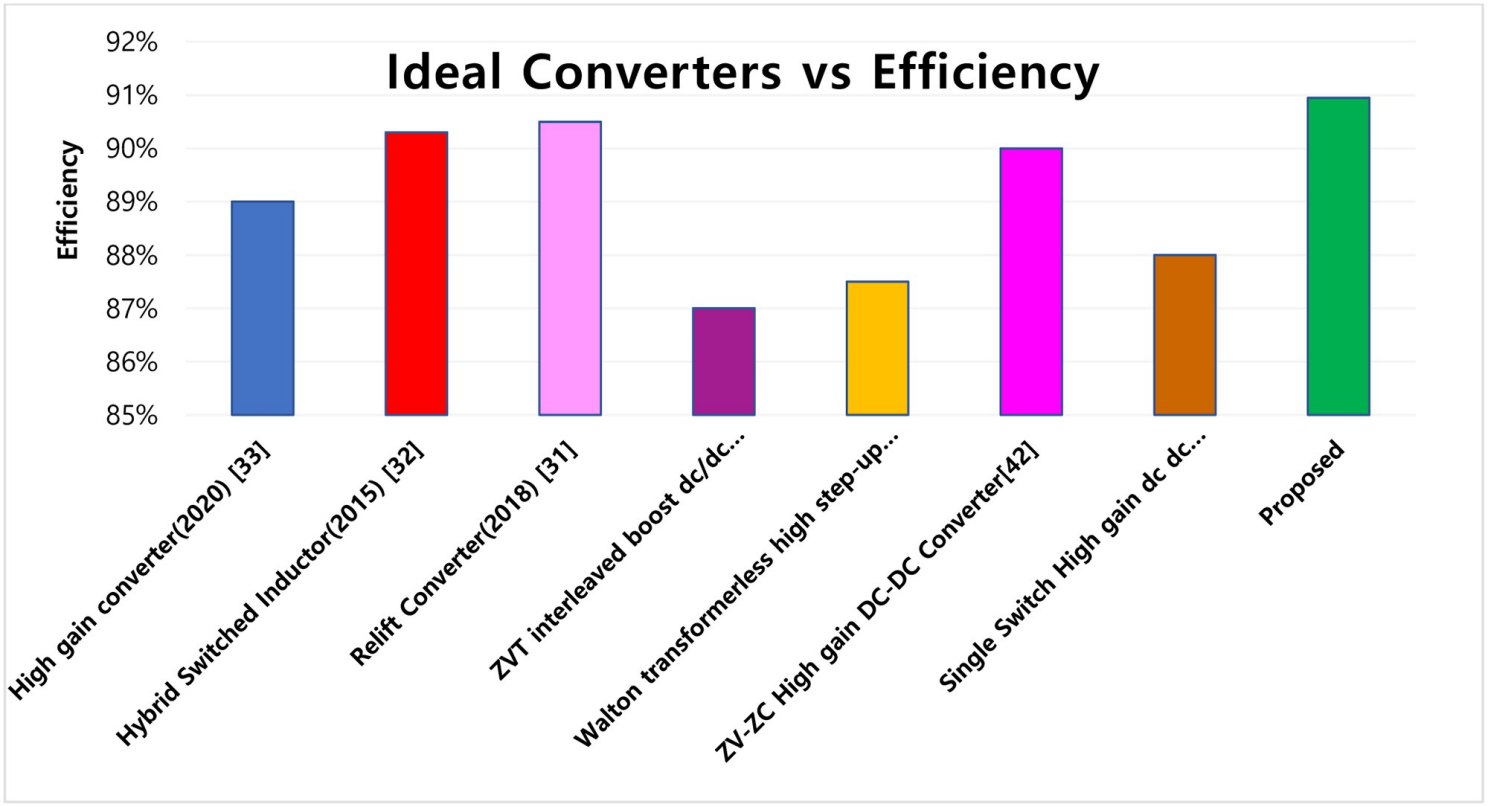
Efficiency comparison.

**Fig 21:**
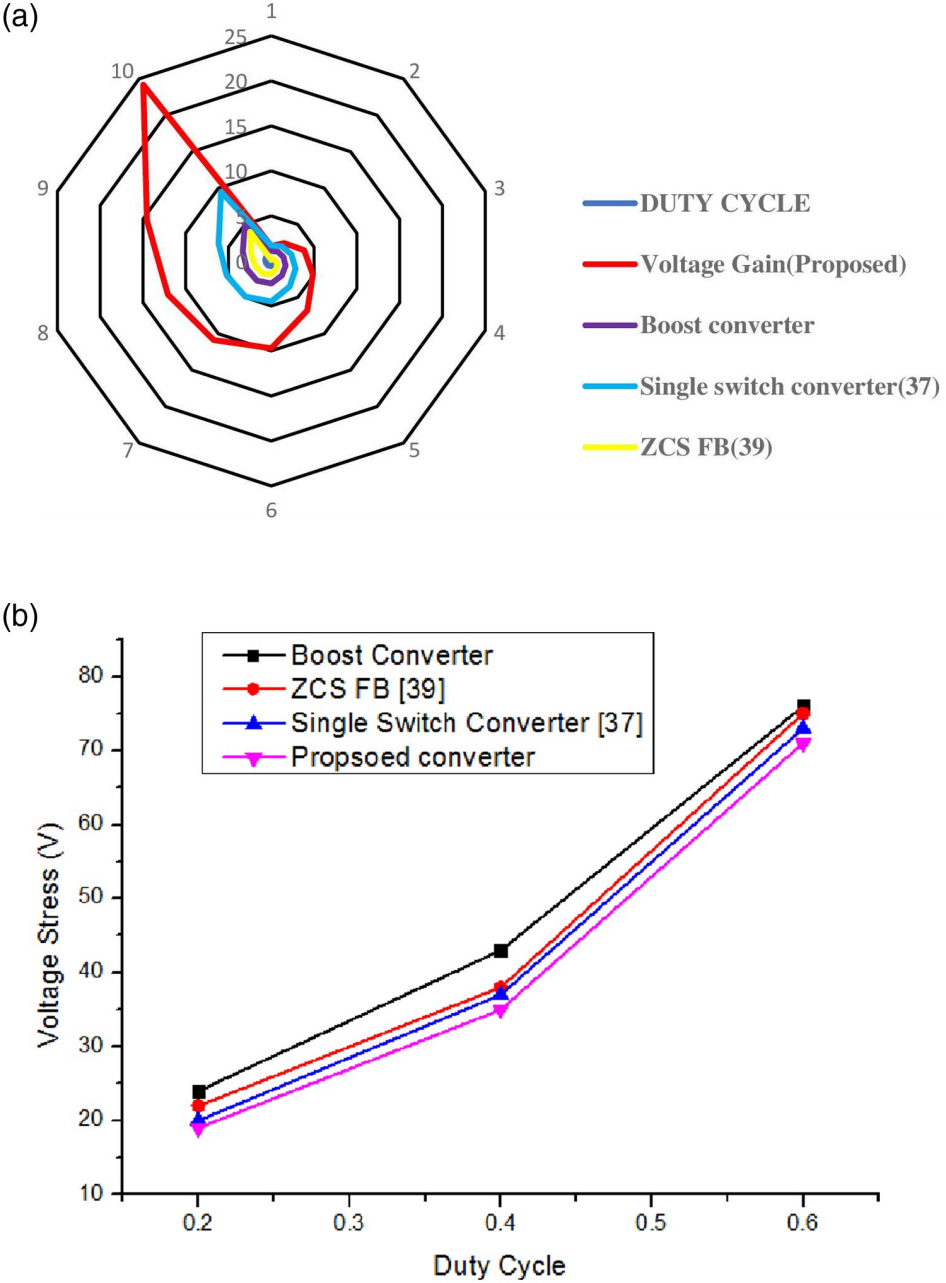
Comparison of voltage gain vs duty cycle and voltages stress across switch vs duty cycle.

In Fig 20 to prove the efficiency of the proposed converter, a comparative analysis has been made with the existing converter topologies. Fig 21 shows the comparison of voltage gain and stress across the switch of the CI based converter.

In comparison to the typical converter, the Proposed converter’s switch is subjected to much less voltage stress, which also aids in the choice of a MOSFET switch with a low rds-on. This lowers switching and conduction losses and hence achieved high conversion efficiency [34, 38]. Table 3 compares the proposed converter with various conventional converters based on the analysis of voltage gain and Duty cycle.

**Table 3:** Duty cycle vs voltage gain.

Duty cycle	Voltage gain (proposed)	Boost converter	Single switch converter (37)	ZCS FB (39)
0.1	1.664	1.11	1.722	0.111
0.2	2.45	1.25	2	0.25
0.3	3.845	1.42	2.35	0.428
0.4	4.866	1.666	2.833	0.6666
0.5	6.8	2	3.5	1
0.6	9.7	2.5	4.5	1.5
0.63	10.875	2.72	4.905	1.702
0.65	12.09	2.857	5.214	1.857
0.7	14.53	3.33	6.166	2.333
0.8	24.2	5	9.5	4
0.9	48.4	10	19.5	9

## Experimental results

**Fig 22:**
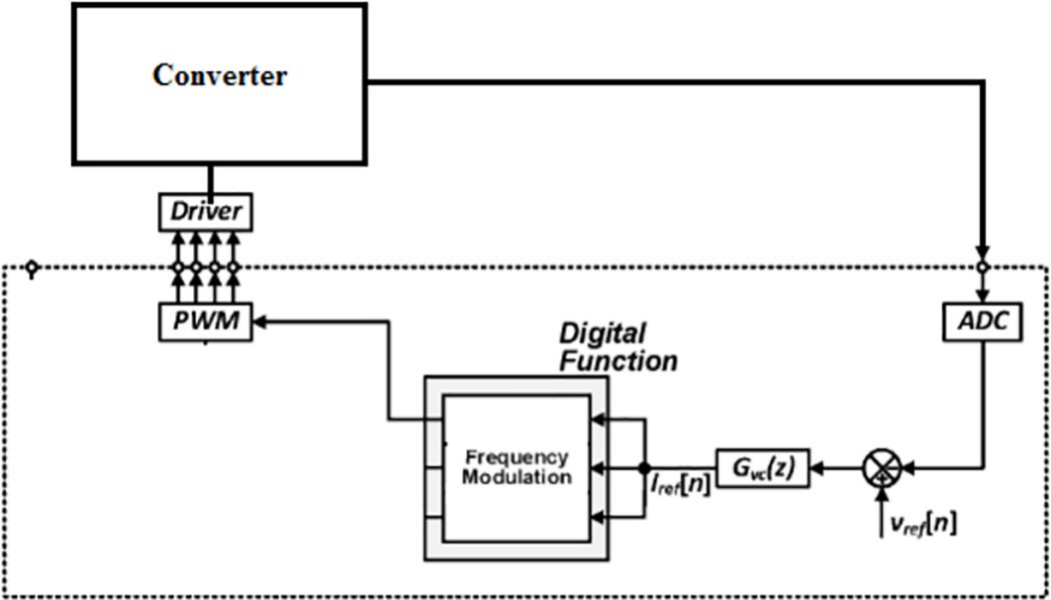
Electrical diagram model of proposed converter.

**Fig 23:**
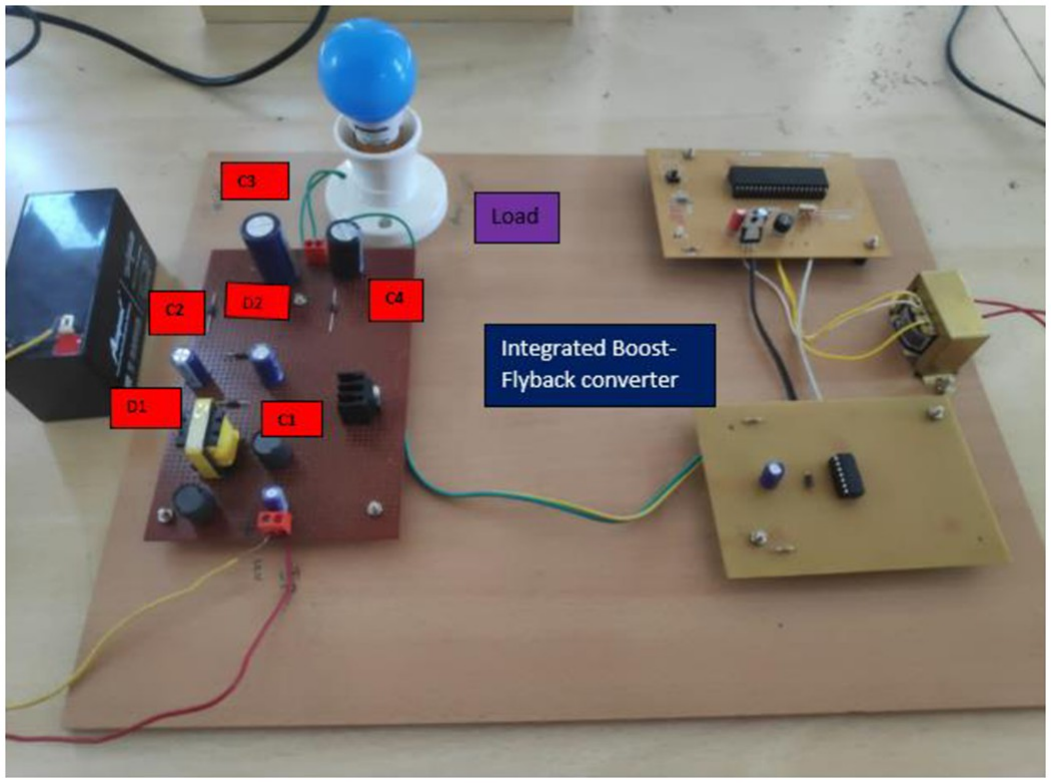
Prototype model of converter.

**Fig 24:**
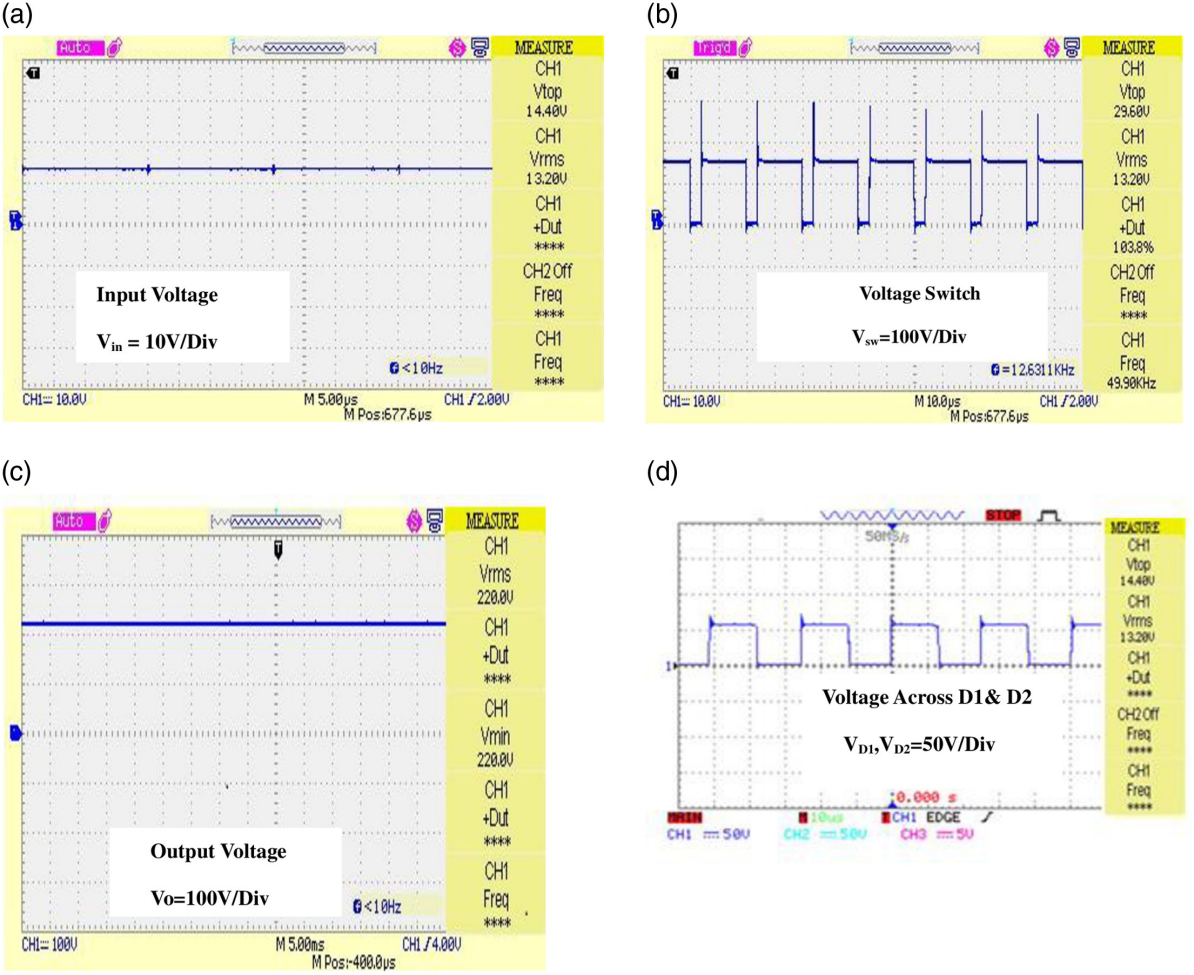
Converter output with controller (A) Input voltage waveform (B) Voltage across the switch (with Controller) (C) Output Voltage (D) Voltage across D_1_ and D_2_.

Fig 22 shows a Electrical diagram model of the proposed converter. Fig 23 depicts the implemented converter in the laboratory setup using FOPID controller. The proposed IBFC Converter is tested for its 18V input voltage and 220V output voltage with 100W in order to validate the theoretical analysis in the CCM mode of operation. The prototype is scaled down and put to the test to show the converter’s superior performance. Fig 24A–24D shows a snapshot of the experimental setup together with the findings and a performance analysis of the converter and controller. The pulse generator operates with a duty cycle of 0.63 and an input voltage of 18V to produce an output voltage of 220V. It has two stages: the boost converter is the first stage, and the flyback converter is the second. A single switch MOSFET powers two stages. The effectiveness of the proposed converter is calculated using the formula (output power/input power) * 100 = 89.75%.

### Performance analysis of the converter with controller

The output of the converter with controller is depicted in Fig 24A–24D. From the figure, it is witnessed that when switch turns ON, resonance occurs with voltage spike. As a result, conduction loss becomes low.

## Conclusion

This paper implements an integrated flyback and boost converter. In comparison to standard integrated DC-DC converter topologies, the proposed converter is modular and takes into fewer number of power devices.

This works advances a single stage dc-dc converter by merging flyback and boost converters by utilising a single switch and also hybrid converter based optimal controller is proposed.

The distinct proposed converters features are as follows

1. The proposed converter is modelled with high elevated gain and less switching stress due to single switch.
2. It comprises the integration of the flyback, boost, and switched network in the flyback converter’s secondary side. The converter’s gain is doubled by the SC.
3. There is reduced switching and conduction loss compared to that of the conventional converter as single switch topology is adopted and ZVS Condition is attained.
4. The results of the experiment and the simulation are both validated to assess the performance of the proposed converter.Based on simulation results, the FOPID Controller is utilised to improve dynamic behaviour and is shown to be effective.
5. A 100W universal hardware prototype has been used to develop, implement, and test the proposed converter. 90.9% is the calculated peak efficiency. The proposed control approach and ideal design are demonstrated by an experimental waveform. The following list highlights the converter’s overall advantages.
  (a) it has a greater voltage gain with higher efficiency
  (b) switching and conduction losses are less

In comparison to current topologies, the proposed IBFC topology results are competitive in terms of cost and efficiency. The proposed converter’s obtained full load efficiency of 90.9% makes it a desirable alternative for PV applications.
